# Do mitochondria play a role in remodelling lace plant leaves during programmed cell death?

**DOI:** 10.1186/1471-2229-11-102

**Published:** 2011-06-06

**Authors:** Christina EN Lord, Jaime N Wertman, Stephanie Lane, Arunika HLAN Gunawardena

**Affiliations:** 1 Department of Biology, Dalhousie University, 1355 Oxford Street, Halifax, B3H 4R2, Canada

## Abstract

**Background:**

Programmed cell death (PCD) is the regulated death of cells within an organism. The lace plant (*Aponogeton madagascariensis*) produces perforations in its leaves through PCD. The leaves of the plant consist of a latticework of longitudinal and transverse veins enclosing areoles. PCD occurs in the cells at the center of these areoles and progresses outwards, stopping approximately five cells from the vasculature. The role of mitochondria during PCD has been recognized in animals; however, it has been less studied during PCD in plants.

**Results:**

The following paper elucidates the role of mitochondrial dynamics during developmentally regulated PCD *in vivo *in *A. madagascariensis*. A single areole within a window stage leaf (PCD is occurring) was divided into three areas based on the progression of PCD; cells that will not undergo PCD (NPCD), cells in early stages of PCD (EPCD), and cells in late stages of PCD (LPCD). Window stage leaves were stained with the mitochondrial dye MitoTracker Red CMXRos and examined. Mitochondrial dynamics were delineated into four categories (M1-M4) based on characteristics including distribution, motility, and membrane potential (ΔΨ_m_). A TUNEL assay showed fragmented nDNA in a gradient over these mitochondrial stages. Chloroplasts and transvacuolar strands were also examined using live cell imaging. The possible importance of mitochondrial permeability transition pore (PTP) formation during PCD was indirectly examined via *in vivo *cyclosporine A (CsA) treatment. This treatment resulted in lace plant leaves with a significantly lower number of perforations compared to controls, and that displayed mitochondrial dynamics similar to that of non-PCD cells.

**Conclusions:**

Results depicted mitochondrial dynamics *in vivo *as PCD progresses within the lace plant, and highlight the correlation of this organelle with other organelles during developmental PCD. To the best of our knowledge, this is the first report of mitochondria and chloroplasts moving on transvacuolar strands to form a ring structure surrounding the nucleus during developmental PCD. Also, for the first time, we have shown the feasibility for the use of CsA in a whole plant system. Overall, our findings implicate the mitochondria as playing a critical and early role in developmentally regulated PCD in the lace plant.

## Background

### Programmed cell death in plants

Programmed cell death (PCD) is the regulated death of a cell within an organism [1]. In plant systems, developmentally regulated PCD is thought to be triggered by internal signals and is considered to be a part of typical development. Examples of developmentally regulated PCD include, but are not limited to, deletion of the embryonic suspensor [2], xylem differentiation [3,4], and leaf morphogenesis [5-12] as is seen in the lace plant (*A. madagascariensis*) and *Monstera obliqua*. The mitochondrion is known to function in PCD in animal systems and the role of the organelle has been largely elucidated within this system; conversely, less is known regarding the mitochondria and PCD in plants [13,14].

### The role of the mitochondria during developmental programmed cell death (PCD)

Within animal systems, mitochondria appear to undergo one of two physiological changes leading to the release of internal membrane space (IMS) proteins, allowing for the membrane permeability transition (MPT), inevitably aiding in PCD signaling. One hypothesized strategy involves the permeability transition pore (PTP), a multi-protein complex consisting of the voltage dependent ion channel (VDAC), the AdNT, and cyclophilin D (CyD) [15]. The formation of the PTP can be initiated by a number of factors including, but not limited to: cell injury [16-18], oxidative stress [15,16], the accumulation of Calcium (Ca^2+^) in the cytosol or mitochondrial matrix [13,19], increases in ATP, ROS, and phosphate, as well changes in pH [20,21]. In addition, evidence suggests that cyclosporine A (CsA) can act in disrupting the PTP by displacing the binding of CyD to AdNT [19] within animal systems. The theory that CsA can inhibit PTP formation has lead to key advances in understanding the second pathway through which mitochondria can release IMS proteins.

The second strategy is proposed to involve the Bcl-2 family of proteins and utilizes only the VDAC. The Bcl-2 family can be divided into two distinct groups based on functionality: the anti-apoptotic proteins including Bcl-2 and Bcl-xL, and the pro-apoptotic proteins including Bax, Bak, Bad and Bid [18,22]. If the amount of pro-apoptotic Bcl-2 proteins increase or the amount of anti-apoptotic Bcl-2 proteins decreases, the VDAC will then work independently to release IMS proteins to aid in PCD signaling.

### The lace plant and programmed cell death

**Figure 1:**
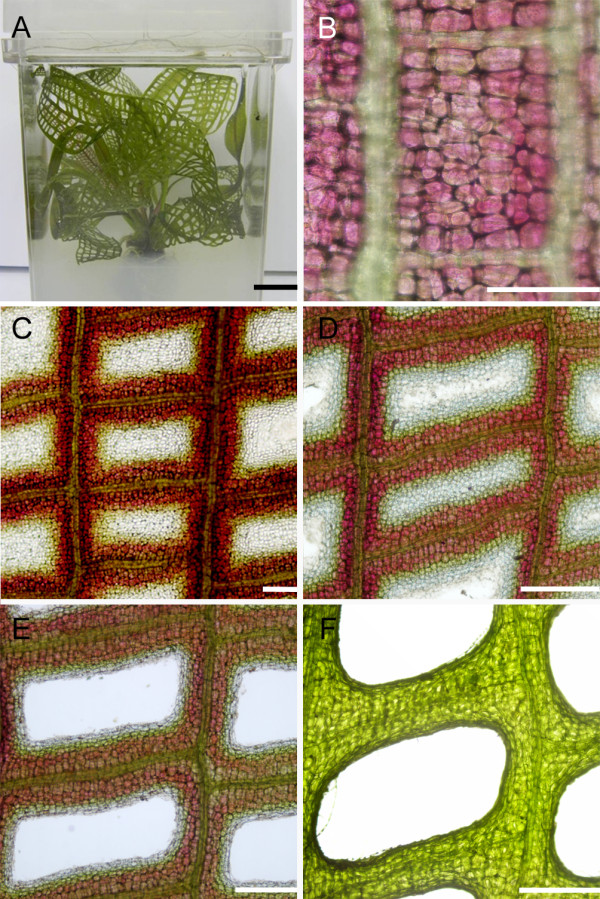
**Progression of developmental PCD within a lace plant leaf, stages (1-5)**. Delineation of leaf morphogenesis in lace plant leaves as PCD progresses. A) Whole plant growing in sterile culture in a magenta box filled with liquid and solid Murashige and Skoog (MS) medium. B) Stage 1, or pre-perforation lace plant leaf, note the abundance of the pink pigment anthocyanin within most cells of the leaf. Also note that one full areole is shown bound by vascular tissue. C) Stage 2, or "window" stage lace plant leaf, note the distinct cleared area in the center of the vasculature tissue indicating a loss of pigments anthocyanin and chlorophyll. D) Stage 3, or perforation formation lace plant leaf. The cells in the center of the cleared window have begun to break away, forming a hole in the center of the areole. E) Stage 4, or perforation expansion lace plant leaf, note that cell death has stopped approximately 4-5 cells from the vascular tissue. F) Stage 5, or a completed perforation in a lace plant leaf. The cells bordering the perforation have transdifferentiated to become epidermal cells. Scale bars (A) = 1 cm; (B) = 200 μm; (C-F) = 500 μm.

The aquatic freshwater lace plant (*A. madagascariensis*) is an excellent model system for the study of developmental PCD in plants. It is one of forty species in the monogeneric family Aponogetonaceae, and is the only species in the family that forms perforations in its leaves via the PCD process [5,7-12]. The leaves of the plant are very thin and transparent, facilitating long-term live cell imaging of the cell death process. A well-developed method for sterile culture of the plant also provides plant material with no microbial contamination (Figure 1A) [5,7-12].

Perforation formation within the plant is also predictable, with perforations consistently forming in areoles of photosynthetic tissue, between longitudinal and transverse veins over the entire leaf surface (Figure 1B). On a whole plant level, leaf development can be divided into five stages (stage 1-5) [5]. Initially, stage 1 (pre-perforation) involves longitudinally rolled, often pink leaves where no perforations are present. This pink coloration is due to the pigment anthocyanin, which is found in the vacuole of the mesophyll cells (Figure 1B). Stage 2 ("window") is characterized by distinct transparent regions in the centre of the vascular tissue, due to the loss of pigments such as chlorophyll and anthocyanin (Figure 1C). Stage 3 (perforation formation) involves the degradation of the cytoplasm and the cell wall of the cell, resulting in the loss of transparent cells in the centre of the window (Figure 1D). Stage 4 (perforation expansion) is characterized by the expansion of the perforation within the areole (Figure 1E). Lastly, stage 5 (complete perforation) results in a completed perforation (Figure 1F) [5]; these tiny perforations will continue to increase in size as the leaf blade grows.

### Organelles involved in developmental programmed cell death (PCD) within lace plant leaves

The mechanisms of developmentally regulated PCD at a cellular level within the lace plant have begun to be elucidated. Common characteristics of PCD have been previously described during leaf morphogenesis in the lace plant and include: the loss of anthocyanin and chlorophyll, chloroplast degradation, cessation of cytoplasmic streaming, increased vesicle formation and plasma membrane blebbing [5,7-10]. Preliminary results indicate indirect evidence for the up-regulation of ETR1 receptors, as well as for the involvement of Caspase 1-like activity during the PCD process in the lace plant (Unpublished). To date, little research has been conducted on transvacuolar strands and no research has been conducted specifically on the mitochondria within this developmentally regulated cell death system [5,7-10].

### Objective

The following paper will aim to elucidate the role of mitochondrial dynamics with relation to other organelles, during developmentally regulated PCD in the novel model species *A. madagascariensis*, using live cell imaging techniques.

## Results

**Figure 2:**
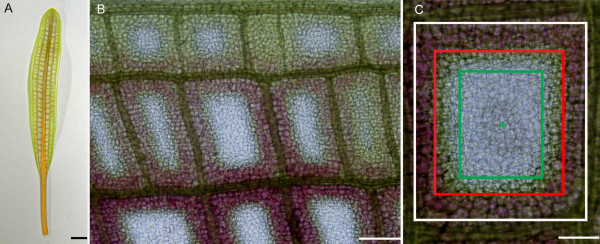
**Description of the PCD gradient within a window stage lace plant leaf**. The three-part differentiation of an areole within a stage 2, or window stage leaf. A) A detached stage 2, or "window" stage leaf. Note the green and pink coloration, which is due to the presence of the pigments chlorophyll and anthocyanin, respectively. B) Single side of a window stage leaf, cut at the midrib. Note the gradient of PCD, in that PCD is most advanced closest to the midrib (bottom) and least advanced towards to leaf edge (top). C) PCD has also been delineated at the level of a single areole. Within a single areole of a stage 2, or window stage leaf, cells closest to the vasculature tissue (between white and red lines) will not undergo PCD and are known as non-PCD cells (NPCD); NPCD cells often contain a marked amount of the pigment anthocyanin. The next group of cells (between red and green lines) are in very early stages of PCD and are known as early PCD cells (EPCD); EPCD cells often contain a marked amount of the pigment chlorophyll. The centermost cells (green lines inward) are cells in late stages of PCD, and are known as late PCD cells (LPCD); LPCD cells have lost most of their pigment, and are clear in nature. Scale bars (A) = 25 mm; (B) = 500 μm; (C) = 250 μm.

Within a stage 2, or window stage leaf (Figure 2A), developmental PCD is least advanced at the leaf blade edge and most advanced closest to the midrib (Figure 2B) [10]. In order to further elucidate organelle changes during PCD, an individual areole within a window stage leaf has been subdivided into three different areas based on the progression of cell death. Non-PCD cells (NPCD; previously regarded as 1b by Wright et al. 2009) line the inside border of a window and consist of cells will never undergo cell death; these cells are normally markedly pink in color due to the pigment anthocyanin. This area is denoted in Figure 2C, and consists of all cells between the white and red lines. The cells adjacent to the NPCD cells will die via PCD, but are in the earliest stages of PCD (EPCD; previously regarded as 2b by Wright et al. 2009). They generally contain no anthocyanin and are green in color due to aggregations of chloroplasts within the cells, sometimes surrounding the nucleus. These cells are denoted in Figure 2C, and consist of all cells between the red and green lines. The next delineation of cells are those found in the center of the window that are at the latest stage of cell death (LPCD; previously regarded as 3b by Wright et al. 2009). These cells are represented in Figure 2C, and consist of cells within the green lines. The presence of these differing stages of PCD within one areole provides a convenient gradient of cell death through which whole leaf observations are facilitated.

### Mitochondrial distribution and motility

Following the determination of optimal dye loading concentrations and incubation time periods, leaf sections were incubated in 0.6 μM MitoTracker Red CMXRos (CMXRos) for 1 hour at room temperature in the dark. Following an intensive rinsing procedure, leaf pieces stained via this method displayed intense mitochondrial staining with little background staining, although it can be noted that a small amount of CMXRos dye is sequestered to the cell wall despite the presence or absence of mitochondria. This staining allowed the distribution of mitochondria to be easily identified within the cells, also permitting for the analysis of changes in mitochondria motility. Analysis of mitochondrial motility was completed by selecting still images from time-lapse videos of single epidermal cells at time 0 sec and 30 sec. Mitochondria at time 0 sec remain red, while mitochondria at time 30 sec were false colored green. These images were then overlaid to provide information on mitochondrial movement.

**Figure 3:**
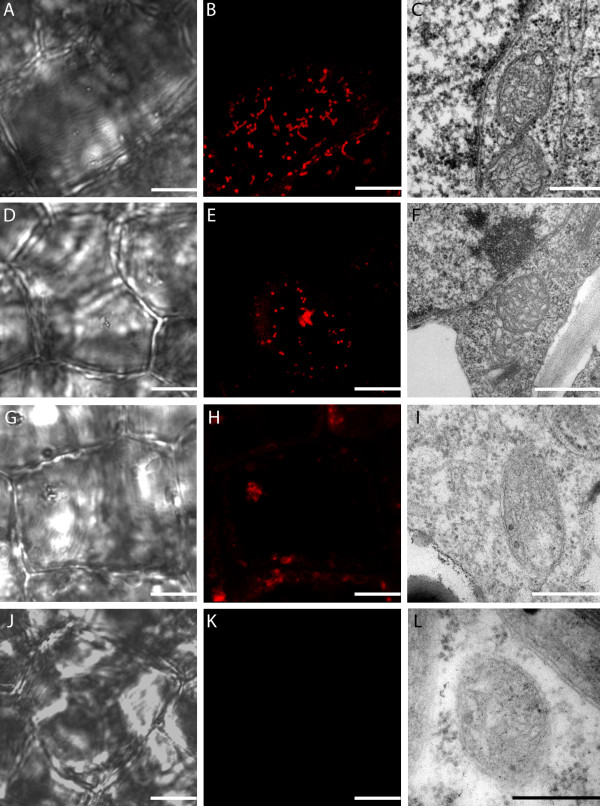
**Mitochondrial distribution (stage M1-M4) within a window stage lace plant leaf**. Mitochondria within a window stage leaf stained with CMXRos and examined via confocal microscopy to view organelle distribution throughout the PCD gradient within individual cells. A) and B) Stage M1 DIC and corresponding CMXRos fluorescent images, respectively. C) TEM micrograph of healthy mitochondria depicting intact mitochondrial membranes and cristae. D) and E) Stage M2 DIC and corresponding CMXRos fluorescent images, respectively. Note mitochondria most have aggregated within the cell with several individual mitochondria still present in the cytosol. F) TEM micrograph of mitochondria within dying cell depicting what appears to be a healthy mitochondria with intact cristae and clear membranes. G) and H) Stage M3 DIC and corresponding CMXRos fluorescent images, respectively. Mitochondria are still aggregated within the cell. I) TEM micrograph of degrading mitochondria, mitochondrial cristae appear to be degraded, with less clear inner and outer membranes as compared to controls. J) and K) Stage M4 DIC and corresponding CMXRos fluorescent images, respectively. Note mitochondria have lost membrane potential entirely and are no longer visible in the fluorescent image. Mitochondria are now considered un-viable. L) TEM micrograph of presumably dead mitochondria depicting nearly indistinguishable membranes and damaged cristae. Scale bars (A, B, D, E, G, H, J, K) = 10 μm; (C, F, I, L) = 0.5 μm

**Figure 4:**
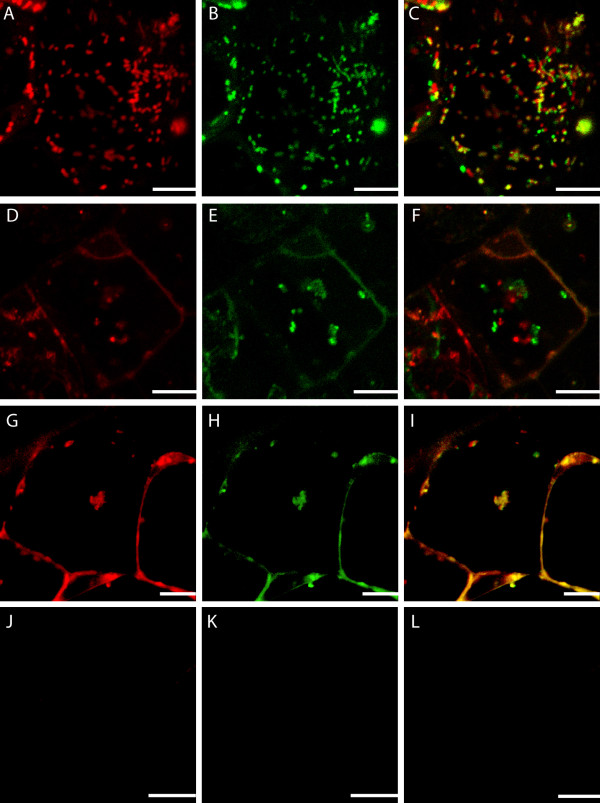
***In vivo *examination of mitochondrial motility and membrane potential in stage M1-M4 mitochondria within a single areole of a window stage lace plant leaf**. Still images selected from time-lapse videos at time 0 and time 30 seconds following CMXRos staining. Mitochondria in time 30 sec images have been false colored green to allow for comparative overlay images to demonstrate mitochondrial motility. A, D, G and J) time 0 seconds CMXRos stained images of M1, M2, M3 and M4 mitochondria over the PCD gradient (NPCD-LPCD), respectively. B, E, H and K) time 30 seconds CMXRos stained images of M1, M2, M3 and M4 mitochondria over the PCD gradient (NPCD-LPCD), respectively. C, F, I and L) Overlay of time 0 and 30 second still images of M1, M2, M3 and M4 mitochondria over the PCD gradient (NPCD-LPCD), respectively. Note that when mitochondria have not moved, overlay images appear yellow. These overlay images characterize the rapid mitochondrial movement of M1 and M2 stage mitochondria, followed by the decrease in mitochondrial motility in M3 and M4 stage mitochondria. Also note the loss of mitochondrial staining in M4 mitochondria, indicating these organelles appear to have undergone a membrane permeability transition and have lost their membrane potential. Still images A, B and C taken from additional file 5. Still images D, E and F taken from additional file 6. Still images G, H and I taken from additional file 7. Still images J, K and L taken from additional file 8. Scale bars (A-I) = 10 μm.

Within a single areole of a stage 2 (window stage) leaf, mitochondrial dynamics were delineated into four categories (M1-M4) based on the gradient of PCD. It is important to note that although these stages are seen simultaneously in a window stage leaf areole, if one was to examine a pre-perforation (stage 1) window, in which no cell death is yet visible, only stage M1 mitochondria would be present (data not shown). Stage M1 mitochondria were consistently found in healthy, NPCD cells (Figure 2C, between white and red lines). These mitochondria were generally seen individually, appeared to have intact membranes and cristae, and illustrated active streaming within the cytosol (Figure 3A, B and 3C; 4A, B and 4C; Table 1; see Additional File 1). Stage M2 mitochondria were generally found within EPCD window stage cells (Figure 2C, between red and green lines), surrounding the interior border of the NPCD cells. These mitochondria were generally seen clustered into several small aggregates, with individual mitochondria in the surrounding cytosol (Figure 3D, E and 3F; Table 1). The movement of stage M2 mitochondrial aggregates (Figure 4D, E and 4F) was more sporadic, random and quicker than M1 stage mitochondria (Figure 4A, B and 4C; see Additional File 2). Stage M3 mitochondria were generally found within LPCD window stage cells (Figure 2C, between green lines and green asterisks). These mitochondria were again seen in aggregate(s) with few to no individual mitochondria within the surrounding cytosol (Figure 3G and 3H). M3 mitochondria begin to display degraded cristae and unclear inner and outer membranes (Figure 3I). Stage M3 mitochondrial aggregates also showed little to no movement as compared to M1 and M2 stage mitochondria (Figure 4A, B, C, D, E, F, G, H and 4I; Table 1; see Additional Files 3 and 4). Lastly, stage M4 mitochondria were also generally located within LPCD cells, but closest to the center of the areole (Figure 2C, denoted by asterisk) and showed absolutely no staining (Figure 3J and 3K). These mitochondria appeared to have dramatically degraded cristae and nearly indistinguishable membranes via TEM imaging and also displayed no movement (Figure 3L; Figure 4J, K and 4L; Table 1; see Additional File 5).

**Table 1:** Mitochondrial stage, distribution, dynamic state, and ΔΨ_m_, as compared to window stage cell staging

Window Leaf Stage	NPCD	EPCD	LPCD	
**Mitochondrial Stage**	M1	M2	M3	M4

**Mitochondrial distribution**	Individual	Aggregates	Aggregates	Aggregates

**Mitochondrial dynamics**	Streaming	Streaming	Cessation of movement	Cessation of movement

**Mitochondrial ΔΨ_m _intactness**	Intact	Intact	Intact	Lost

### Decrease in mitochondrial ΔΨ_m_

Window stage leaf pieces stained with CMXRos were also used to make inferences regarding mitochondrial ΔΨ_m _during developmentally regulated PCD. A reduction in ΔΨ_m _is hypothesized to allow subsequent release of IMS proteins and the continuation of PCD signaling. This shift in ΔΨ_m _can be visualized via changes in CMXRos fluorescence. Stage M1-M3 mitochondria displayed vivid CMXRos staining, providing indirect evidence of the intact ΔΨ_m _(Figure 4A, B, C, D, E, F, G, H and 4I; Table 1). Stage M4 mitochondria showed little to no mitochondrial staining, and are thus expected to have undergone the MPT (Figure 4J, K and 4L; Table 1). It should be noted that despite the lack of mitochondrial fluorescence in M4 stage mitochondria, a ruptured inner or outer mitochondrial membrane was not observed.

### Terminal deoxynucleotidyl transferase mediated dUTP nick-end labeling (TUNEL)

**Figure 5:**
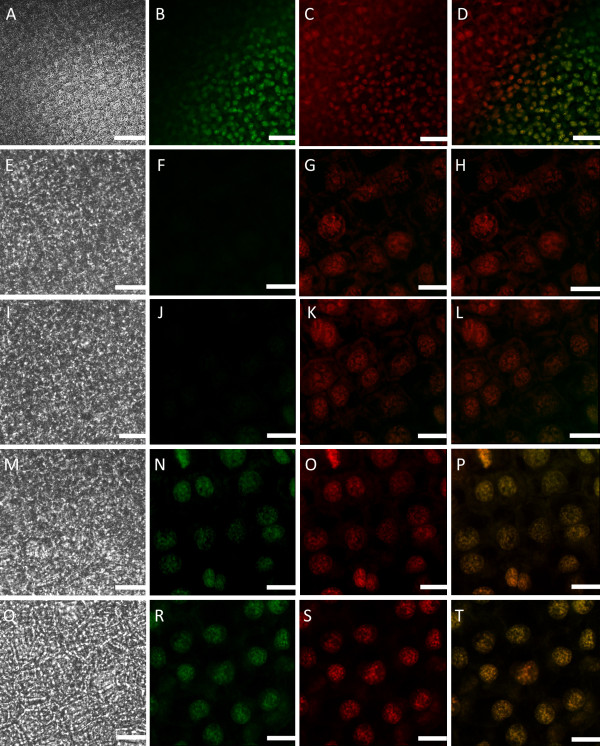
**TUNEL assay portraying TUNEL-positive nuclei within a single areole of a stage 2 or window stage leaf**. TUNEL-positive nuclei within a single areole of a stage 2 (window stage) leaf. Note that Propidium Iodide (PI) staining is red, TUNEL-positive nuclei stain green and when red and green nuclei overlap they appear yellow. A) Low magnification differential interference contrast (DIC) image of a portion of a single areole in a window stage leaf B) Corresponding low magnification TUNEL-positive image C) corresponding low magnification PI image D) overlay of TUNEL-positive and PI images. E-H) High magnification images taken of NPCD cells where stage M1 mitochondria are normally found, DIC, TUNEL assay, PI and overlay of all three respectively. I-L) High magnification images taken of EPCD cells where stage M2 mitochondria are normally found, DIC, TUNEL assay, PI and overlay of all three respectively. M-P) High magnification images taken of LPCD cells where stage M3 mitochondria are normally found, DIC, TUNEL assay, PI and overlay of all three respectively. Q-T) High magnification images taken of LPCD cells where stage M4 mitochondria are normally found, DIC, TUNEL assay, PI and overlay of all three respectively. Scale bars (A-D) = 60 μm; (E-T) = 15 μm.

Further analysis of mitochondrial dynamics during developmentally regulated PCD was completed by the execution of a TUNEL assay and counter staining with propidium iodide (PI) to aid in co-localization (Figure 5). Previously it has been shown TUNEL-positive nuclei are present in stages 2-4 (window stage to perforation expansion) of leaf development [5]. When examining a single areole within a stage 2 (window stage) leaf, there appeared to be a gradient of TUNEL-positive nuclei that corresponded with the progression of mitochondrial death (Figure 5A, B, C and 5D). NPCD cells that contained M1 stage mitochondria showed no TUNEL-positive nuclei (Figure 5E, F, G and 5H). EPCD cells that contained M2 stage mitochondria also contained no TUNEL-positive nuclei (Figure 5I, J, K and 5L). LPCD cells that contained M3 stage mitochondria showed TUNEL-positive nuclei (Figure 5M, N, O and 5P). LPCD cells that contained M4 stage mitochondria consistently showed intense TUNEL-positive staining (Figure 5Q, R, S and 5T).

### Mitochondrial movement and transvacuolar strands

**Figure 6:**
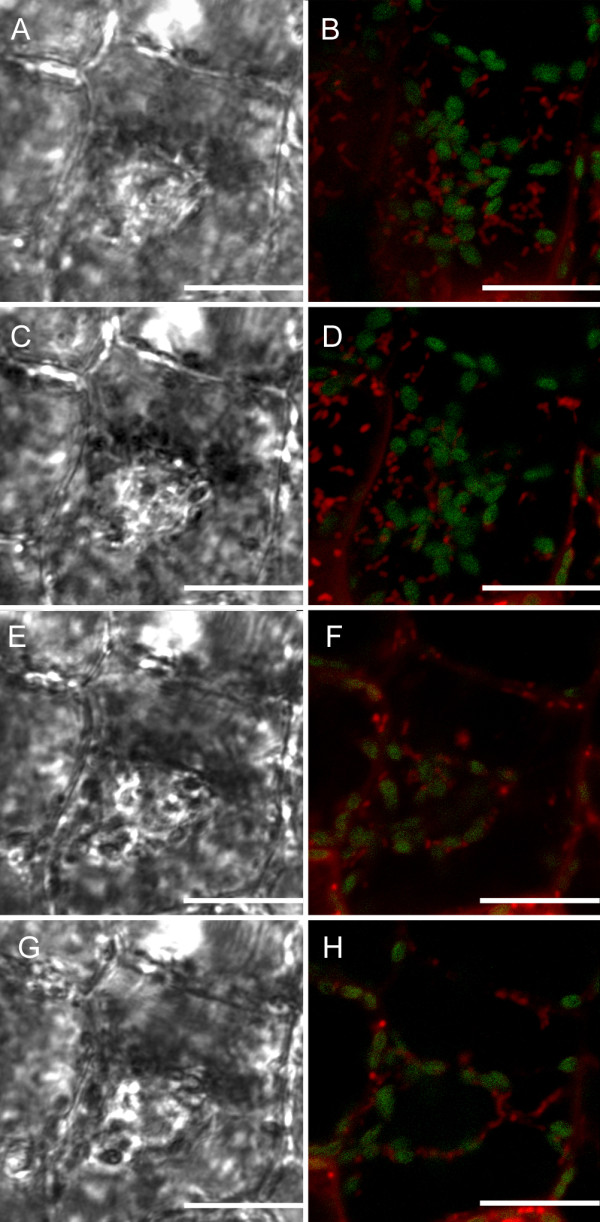
**Progressive Z-stack series of a single cell, illustrating mitochondria and chloroplasts associations with transvacuolar strands within a lace plant window stage leaf**. A z-stack progression consisting of four focal planes within one CMXRos stained cell in the center of a window stage leaf areole. Red fluorescence represents mitochondria while green fluorescence represents chlorophyll autofluorescence. A) and B) DIC and corresponding fluorescent images, respectively, in the top most plane of the cell. Note the mitochondria and chloroplasts around the nucleus. C) and D) DIC and corresponding fluorescent images, respectively in a lower focal plane. E) and F) DIC and corresponding fluorescent images, respectively in a middle focal plane within the cell. Note the continued association of chloroplasts and mitochondria around the nucleus, and the appearance of a strand in the lower right hand corner of the cell. G) and H) DIC and corresponding fluorescent images, respectively, displaying the lower most focal plane within this cell. Note the transvacuolar strand, which appears to have CMXRos stained mitochondria associated with it. Scale bars (A-H) = 25 μm.

**Figure 7:**
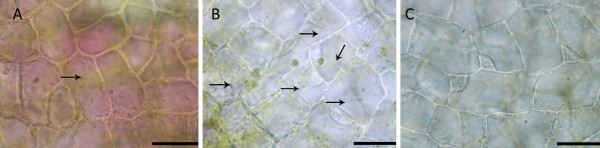
**Light micrographs of NPCD, EPCD and LPCD stage cells illustrating variation in transvacuolar strand activity**. A) NPCD stage cells depicting several transvacuolar strands (black arrow), in which mitochondria and chloroplasts appeared to be associated (see additional file 6) B) EPCD stage cells showing an increase in the number of transvacuolar strands (black arrow) and continued associations with mitochondria and chloroplasts. Depending on the focal plane of the cell, transvacuolar strands appear to be connected with the cell periphery and with the nucleus (see additional file 7). C) LPCD stage cells illustrating a decrease in the number of transvacuolar strands with no organelle affiliations (see additional file 8).

Our results indicate that mitochondria, as well as associated chloroplasts, appear to be moving on transvacuolar strands (Figure 6, see Additional Files 6, 7, 8), possibly allowing for more rapid and organized movements within the cell. Figure 6 illustrates still images taken from a successive Z-stack progression through an EPCD stage single cell. Mitochondria and chloroplasts appear to have distinct associations with one another, and in most instances appear to be congregated around the nucleus (Figure 6A, B, C and 6D). These images also illustrate both mitochondria and chloroplasts moving in clear lines with a trajectory towards the nucleus, along what appears to be transvacuolar strands (Figure 6E, F, G and 6H). At this stage the cells are still healthy and do not show any sign of plasma membrane shrinkage. Transvacuolar strands were examined in NPCD, EPCD and LPCD window stage leaf cells. There appeared to be several transvacuolar strands present in NPCD cells (Figure 7A, Additional File 6), an increase in transvacuolar strand occurrence in EPCD cells (Figure 7B, Additional File 7) and a dramatic decrease in transvacuolar strands in LPCD cells (Figure 7C, Additional File 8).

### Cyclosporine A treatment

#### Qualitative analysis

**Figure 8:**
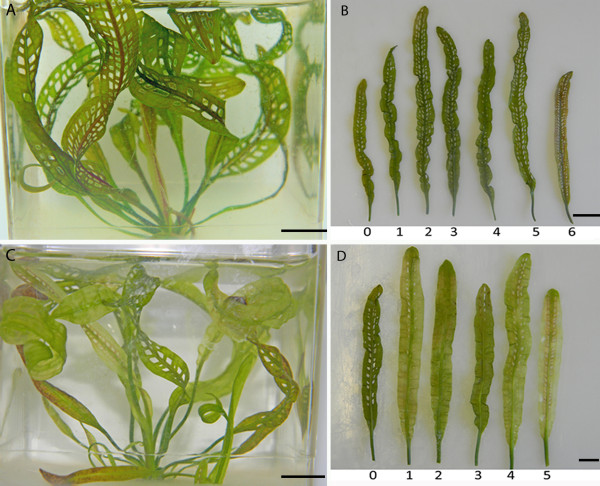
**Qualitative analysis of the effect of CsA, a mitochondrial PTP antagonist on lace plant PCD**. Representative digital images of whole lace plants in magenta boxes, and harvested leaves, in the order of their emergence, from the corresponding box formed during each CsA experiment. A) and B) whole lace plant and corresponding leaf harvest for control plants, respectively; C) and D) whole lace plant and corresponding leaf harvest for 10 μM controls, respectively. For all harvested leaf images, leaves are arranged in chronological order of formation with leaf 0 representing a leaf formed prior to the initiation of the experiment, and subsequent leaves 1 through 4, 5, or 6 having formed after the initiation of the experiment. Note that inhibition of perforation formation is primarily visible for leaves 1-3 for CsA treated plants (C-D). All scale bars = 1 cm.

Figure 8 illustrates the effect of the optimal concentration of CsA (10 μM) on *in vivo *perforation formation within the lace plant. Photographs of boxed plants and harvested leaves of control (just ethanol), and CsA (10 μM) treated plants clearly display a decrease in perforation formation (Figure 8A, B, C and 8D). Concentrations of 2 μM, 4 μM, 15 μM, and 20 μM CsA were also examined (data not shown), with 10 μM being chosen as the minimum concentration to statistically reduce perforation number and not cause a toxic effect. The 20 μM treatment was considered toxic and was not included within the remainder of experiments. The effect of CsA seemed to dissipate following the growth of three new leaves from the SAM, indicating initial rapid uptake of CsA or possibly a rapid disintegration of CsA overtime (Figure 8).

#### Quantitative analysis

**Figure 9:**
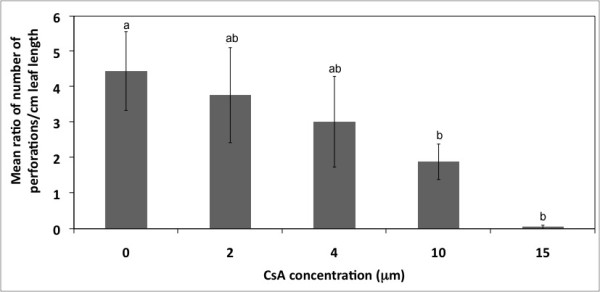
**Quantitative analysis of the effect of CsA, a mitochondrial PTP antagonist on lace plant PCD**. The effect of CsA on the mean ratio of number of perforations per cm of leaf length for control and treatment groups. The mean ratio of number of perforations per cm of leaf length decreased with increasing concentrations of CsA, indicating that the inhibition of the PTP via CsA reduced the amount of PCD occurring in lace plant leaves. Significant relationships were found between the control, 10 μM and 15 μM treatment groups (P < 0.05). No significant relationships were found between the treatment groups (P > 0.05). Number of leaves per control and treatment group ranged from n = 30-60. Bars with the same letters are not significantly different.

The GLM ANOVA revealed significant differences in the ratio of number of perforations per cm of leaf length between the CsA treated plants at 10 μM (P = 0.0035) and 15 μM (P = 0.0007) compared to control plants (P < 0.05; Figure 9). There was no significant difference in the ratio of number of perforations per cm of leaf length between CsA treated plants at 2 μM (P = 0.1572) and 4 μM (P = 0.0545) compared to control plants (P > 0.05; Figure 9). CsA treatments at 2 μM and 4 μM differed significantly from CsA treatments at 10 μM and 15 μM (P < 0.05). The analysis also revealed that there was no overall significant difference in leaf length between control and any CsA treated plants (P > 0.05).

### Mitochondrial dynamics following CsA treatment

**Figure 10:**
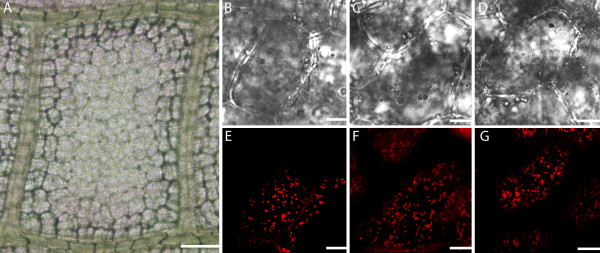
***In vivo *examination of mitochondrial distribution following pre treatment with the mitochondrial PTP inhibitor CsA**. Changes in mitochondrial dynamics within one areole examining the same cellular areas (NPCD-LPCD) as observed within control window stage leaves. A) A single areole within a 10 μM CsA treated leaf 4 days following its emergence from the SAM. B and E) DIC and CMXRos images of a cell that corresponds with an NPCD window stage cell, respectively. C and F) DIC and CMXRos images of a cell that corresponds with an EPCD window stage cell, respectively. D and G) DIC and CMXRos images of a cell that corresponds with an LPCD window stage cell, respectively. Note even distribution of mitochondria at each stage. Scale bars (A) = 100 μm; (B and G) = 10 μm.

**Figure 11:**
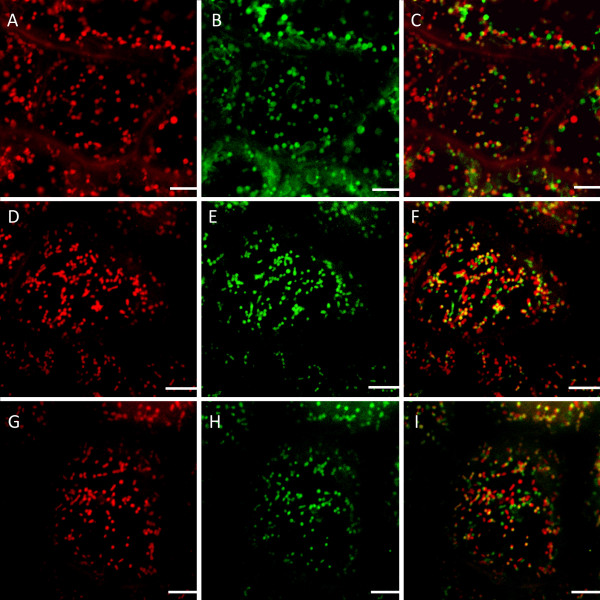
***In vivo *examination of mitochondrial motility and membrane potential following pre treatment with the mitochondrial PTP inhibitor CsA**. Still images selected from time-lapse videos at time 0 and time 30 seconds following 10 μM CsA treatment and subsequent CMXRos staining. Mitochondria in time 30 seconds images have been false colored green to allow for comparative overlay images to demonstrate mitochondrial motility. Note that when the red and green overlap, the mitochondria appear yellow and are presumably still. A, D, G) time 0 seconds CMXRos stained images of CsA treated leaves corresponding with NPCD, EPCD and LPCD cells, respectively. B, E, H, K) time 30 seconds CMXRos stained images of CsA treated leaves corresponding with NPCD, EPCD and LPCD cells, respectively. C, F, I) Overlay of time 0 and 30 second still images corresponding with NPCD, EPCD and LPCD cells, respectively. These overlay images characterize the rapid mitochondrial movement in CsA treated leaves. Still images A, B and C taken from additional file 9. Still images D, E and F taken from additional file 10. Still images G, H and I taken from additional file 11. Scale bars (A-I) = 10 μm.

Following the conclusion that 10 μM was the optimal concentration to prevent PCD and perforation formation within the lace plant, CsA treated leaves were stained with CMXRos to examine mitochondrial dynamics (Figure 10 and 11). Mitochondrial dynamics were again examined within one areole, between vascular tissue, where PCD would have occurred in control leaves. Mitochondria were examined in areas that would be equal to NPCD, EPCD and LPCD areas within a control window stage leaf. CsA treated mitochondria appeared to remain individual, rounded, and evenly distributed from NPCD-LPCD cellular areas (Figure 10B, C, D, E, F and 10G). Several small aggregates did appear in some LPCD cells, but were not consistent in every cell. The mitochondria also appeared to remain actively streaming in the cytosol, and showed no loss of membrane potential within similar cellular areas examined within window stage leaves (NPCD-LPCD; Figure 11A, B, C, D, E, F,G. H and 11I; see Additional Files 9, 10 and 11)

## Discussion

### Developmentally regulated programmed cell death

The unique and predictable system of developmentally regulated PCD within the lace plant offers an excellent model for the study of organelle changes during this process. Within this study, we showed the importance of the mitochondria within the early stages of PCD. In addition, we have illustrated the possible strong correlation between the mitochondria and other organelles including the chloroplasts, nuclei and transvacuolar strands.

### Variation in mitochondrial distribution, dynamics and ΔΨm

The observation that the chloroplasts formed a ring formation around the nucleus in the lace plant in the mid to late stages of PCD has been reported previously by Wright et al. (2009) [10]; however, this is the first report of the association of mitochondria with these chloroplasts surrounding the nucleus. The reasons for the above are not known, however, it is possible they congregate due to a structure-function relationship, to aid in the PCD process. Given the active movement of mitochondria and chloroplasts on transvacuolar strands towards the nucleus, as seen through live cell imaging (See Additional File 7) we can confirm that this association is not due to plasma membrane shrinkage, given none is present. A phenomenon noted in cucumber, pea and rye plants following induced cell death with ethylene illustrated that mitochondria located in parenchyma cells were attracted to the nuclear envelope during PCD. Authors reported that this attraction led to the condensation of chromatin at the sites where the organelles were in contact, and was thus considered to be a structural mechanism for PCD promotion [23].

The aggregation of mitochondria appears to be the first visible shift in mitochondrial dynamics during developmentally regulated PCD in the lace plant. This aggregation of mitochondria has also been demonstrated during induced cell death systems by Scott and Logan (2008) [24], Yao et al. (2004) [17] and Gao et al. (2008) [25] in *Arabidopsis *protoplasts, and also by Lord and Gunawardena (2011) [26] in lace plant protoplasts. The reason for the formation of aggregates is unknown. Previous studies report that these mitochondrial aggregates during PCD in *Arabidopsis *[25], lace plant [26] and tobacco protoplasts [27] are located in the cytosol of the cells. However, recent data (unpublished) from the Gunawardena lab suggest that this aggregate may be inside the vacuole at later stages of PCD. These recent findings, along with the rapid and random movements of the aggregate, suggest that this aggregate may move from the cytosol to the vacuole during late PCD, possibly to be degraded. Also, this study never observed the aggregates moving along TVS, suggesting that they may be in the vacuole at this time. Previous studies by Wright et al., 2009 [10] in developmental PCD in the lace plant provide evidence of similar aggregates, containing chloroplasts and possibly mitochondria, inside the vacuole undergoing Brownian motion during the later stages of PCD (see Supplementary Video 6 in [10]). However, whether these aggregates are first in the thin layer of cytoplasm and then move into the vacuole requires further investigation.

Following aggregation, mitochondria displayed a subsequent reduction in streaming. This cessation of streaming has also been demonstrated in mitochondria during several induced cell death examples in *Arabidopsis *protoplasts [16,25], *Arabidopsis *leaf discs [28], tobacco BY-2 cells [29,30], and lace plant protoplasts [26]. This impairment of mitochondrial movement is commonly seen following the induction of cell death, and is thought to be highly correlated with the acute change in cellular redox status, as well as the remainder of the cell death process [29,30,16].

Following mitochondrial aggregation and cessation of streaming, they appear to undergo the MPT, characterized by a loss of CMXRos staining. The decrease in ΔΨ_m _appeared to occur between M3 and M4 mitochondria, possibly indicating that this is the first visible indication of membrane transition, and thus, possibly the first release of IMS proteins. The release of these IMS components at this time would correlate with the apparent degradation of the inner mitochondrial structure at this stage of PCD. This decrease in mitochondrial ΔΨ_m _has been noted as a key characteristic of cell death in animal systems, and has also been demonstrated in a variety of other plant examples including induced cell death in *Arabidopsis *protoplasts [17,24,25], isolated oat mitochondria [31], lace plant protoplasts [26] and also during developmentally regulated cell death in isolated Zinnia treachery element (TE) cells [32].

### Terminal deoxynucleotidyl transferase mediated dUTP nick-end labeling (TUNEL)

A trend was noted within a single areole of a stage 2 (window stage) leaf; cells that contained TUNEL-positive nuclei were generally correlated with cells that contained M3 and M4 stage mitochondria. TUNEL-positive nuclei were not seen in NPCD cells that contained stage M1 mitochondria; this result was expected given that these cells are not pre-disposed to undergo cell death. TUNEL-positive nuclei were also absent in EPCD cells that contain stage M2 mitochondrial aggregates. This result clearly indicates that mitochondrial changes have begun prior to the fragmentation of nuclear DNA leading to TUNEL-positive nuclei. TUNEL-positive nuclei were consistently seen within LPCD cells that contained either M3 or M4 stage mitochondria. This trend also allows us to conclude that mitochondrial changes, particularly those seen in stage M3 and M4 stage mitochondria, including the cessation of mitochondrial movement and complete loss of ΔΨ_m _are probably occurring simultaneously with the fragmentation of nuclear DNA, as noted by the presence of TUNEL-positive nuclei within these areas.

### Transvacuolar strands

An increased number of transvacuolar strands was noted in window stage leaf cells that were in the early stages of PCD (EPCD cells). This increased instance of transvacuolar strands is a common characteristic of PCD and has been noted previously during developmental cell death in the lace plant [10], in induced cell death in lace plant protoplasts [26], and also during induced cell death by osmotic stress in tobacco suspension cultures [33]. Increases in transvacuolar strands could aid in the movement of organelles such as chloroplasts and mitochondria within plant cells. Within this system, both of these organelles have been seen traveling along thin strands spanning the vacuole of the cell and sometimes appearing to be moving towards the nucleus. The appearance of these strands decreases as PCD progresses, with few to no transvacuolar strands present in LPCD stage cells.

### Cyclosporin A

The application of the PTP agonist CsA to the lace plant system marks the first time, to our knowledge, that this inhibitor has ever been applied *in vivo*. The inhibitor has been previously used during induced cell death examples on cell cultures [34,35], isolated protoplasts [27,17,24,26], and isolated mitochondria [20,36-39]. The only other developmentally regulated PCD example in which CsA had been employed was during TE differentiation in *Zinnia*, but this example is considered *in vitro *due to the cells being isolated from the plant prior to CsA treatment [32].

The application of CsA to lace plants in magenta boxes led to a reduction in perforation formation in leaves produced following the addition of the inhibitor. This significant decrease in perforation formation within the lace plant via CsA application indirectly indicates that the PTP pathway may play a role in cellular death within this system. Although the involvement of the PTP in animal PCD is well supported, it is controversial if a similar complex has a role in the release of IMS proteins in plant PCD, as shown by the following authors. Studies examining tobacco protoplasts [27], sycamore cells [34] or mitochondria isolated from either winter wheat [38] or potato tubers [20], as well as developing tracheary elements [32] provide evidence suggesting that CsA effectively inhibits or delays PCD; this, arguably, suggests a role for the PTP in plant PCD. However, there have also been studies that demonstrate the insensitivity of plant PCD to CsA [39]. Lin et al., 2006 report a delay or reduction in PCD, and suggest that this may provide evidence for the alternate pathway. In animal systems, there is an alternate pathway for the release of IMS proteins that involves the Bcl-2 family of proteins, however, to date there is no direct evidence of Bcl-2 family proteins in plants. Inhibitor experiments, however, provide indirect evidence for Bcl-2-like family protein activity in plants [40]. In contrast to this study, our experiment reports a significant reduction in PCD following CsA pre-treatment, suggesting the absence of an alternative pathway in this system. This provides indirect evidence for the role played by the PTP in lace plant PCD. However, further studies are required to examine the role of the PTP in the release of the IMS proteins from the mitochondria into the cytosol.

CsA concentrations in the lower range (2 μM and 4 μM) did not result in a significantly lower amount of perforations as compared to the controls (data not shown). This observation was expected, given that the inhibitor is dissolved in liquid and being applied to whole plants; therefore, higher concentrations may be required in order to affect the PTP. CsA at 10 μM significantly reduced the amount of perforations in the lace plant as compared to the control, but also maintained a healthy leaf appearance. The observations that no perforations formed at the 15 μM concentration, but did form in the controls, and that some transient leaf clearing occurred, indicates that this may be the lower limit of toxicity for CsA in the lace plant. The 20 μM CsA treatment resulted in brown and/or cleared leaves and therefore this concentration was considered very toxic and was not included in the subsequent statistical analysis. Overall, for further research it has been concluded that 10 μM CsA is the ideal concentration to inhibit the opening of the PTP during lace plant developmentally regulated PCD. This concentration has also been utilized as an optimal concentration in other plant examples, including sycamore cells [34].

For this reason, 10 μM CsA treated leaves, four days following their emergence from the SAM, were chosen for examination; these leaves were therefore a similar developmental age as window stage leaves examined previously. CsA treated leaves depicted numerous, round mitochondria, which generally remained individual within the cytosol, and formed few aggregates in all cell types equal to NPCD-LPCD. These mitochondria also remained streaming within the entire areole and did not appear to undergo a membrane permeability transition causing loss of membrane potential and CMXRos staining. Given that treatment with CsA is hypothesized to block the release of IMS proteins from the mitochondria, we would anticipate variations in mitochondrial dynamics within this system. Intense mitochondrial fluorescence was anticipated, as this drug is hypothesized to inhibit the PTP and possibly the subsequent MPT. A round, and or swollen appearance of mitochondria following CsA treatment was also noted, although the reason behind this is unknown and needs to be further investigated. Overall CsA treated mitochondria display characteristics that most closely resemble M1 mitochondrial dynamics, where no PCD is occurring.

## Conclusions

The results presented here elucidate organelle dynamics, focused on mitochondria, during developmentally regulated PCD in the lace plant *A. madagascariensis*. Developing leaves in which PCD was initiated (window stage) were stained with the mitochondrial membrane potential sensitive dye CMXRos and were examined via live cell imaging and confocal fluorescent microscopy. Observations of mitochondrial aggregation, motility and ΔΨ_m _lead to the classification of mitochondria into one of four stages (M1-M4) based on their location in a window stage leaf areole. Our findings also indicate that within a single areole of a stage 2 (window stage) leaf a gradient of TUNEL-positive nuclei staining exists. TUNEL-positive nuclei were not seen in cells containing M1 and M2 stage mitochondria and were seen in cells with M3 and M4 stage mitochondria. These correlations suggest that the mitochondrial aggregation occurs prior to DNA fragmentation, whereas cessation of mitochondrial streaming and the membrane permeability transition resulting in complete loss of ΔΨm, based on CMXRos staining, probably occurs concurrently with the fragmentation of nuclear DNA. Mitochondria and chloroplasts were examined via live cell imaging, highlighting the role of transvacuolar strands in the movement of the organelles into a ring formation around the nucleus. The function of the mitochondrial PTP during PCD in developing lace plant leaves was also indirectly examined via CsA pre-treatment. Examination of CsA treated mitochondria revealed individual organelles, continued mitochondrial streaming and no loss in membrane potential over the same cellular areas (NPCD-LPCD) within one areole. Overall, results presented here detail organelle dynamics during developmentally regulated PCD in whole lace plant tissue and suggest that the mitochondria plays an important role in the early stages of PCD.

## Methods

### Plant materials

Lace plants used for all experimental purposes were grown in sterile culture in magenta boxes and were maintained via subculture as described by Gunawardena et al. (2006; Figure 1a). Plants were grown with 12 h light/12 h dark cycles provided by daylight simulating fluorescent bulbs (Philips, Daylight Deluxe, F40T12/DX, Markham, Ontario) at approximately 125 μmol·m^-2^·s^-1 ^at 23.5°C. All chemicals were purchased from Sigma (St. Louis, MO, USA), unless otherwise stated. All experiments were completed at least three times unless otherwise stated.

### Light Microscopy

Images of various leaf stages were taken using differential interference contrast (DIC) optics and an eclipse 90i compound microscope (Nikon Canada, Mississauga, Ontario, Canada) fitted with a digital camera (Nikon DXM 1200c) and using NIS Elements imaging and analysis software. This microscope was also used to acquire several of the additional file live cell imaging videos, all of which are in real time unless otherwise stated.

### Confocal laser scanning microscopy

Confocal observations were performed using a Nikon Eclipse T*i *confocal microscope (Nikon, Canada, Mississauga, Ontario, Canada) fitted with a digital camera (Nikon DS-Fi1) and using EZ-C1 3.80 imaging software and Ti-Control. Confocal microscope observations were performed using DIC optics with complimentary fluorescent images taken via a fluorescein isothiocyanate (FITC; excitation 460-500 nm emission 510-560 nm) or Tetramethyl Rhodamine Iso-Thiocyanate (TRITC; excitation 527-552 nm emission 577-632 nm) laser. False color images were prepared by generating still images selected from time-lapse videos at time 0 and time 30 seconds following CMXRos staining. Mitochondria in time 30 sec images were then false colored green and overlaid onto time 0 images to demonstrate mitochondrial motility. This microscope was also used to acquire several of the additional file live cell imaging videos, all of which are in real time unless otherwise stated. All composite plates were assembled using Adobe Photoshop Elements version 6.0.

### Transmission Electron Microscopy

Tissue pieces approximately 2 mm^2 ^were excised from window stage leaves and fixed in 2% glutaraldehyde in 0.05 M sodium cacodylate buffer, pH 6.9, for 24 hours in a vacuum (20 psi). Following overnight incubation, samples were rinsed in buffer and post fixed in 2.5% aqueous osmium tetroxide for 4 h at room temperature. Tissues were then dehydrated in a graded ethanol series, and placed through ethanol:Spurr resin mixtures. Tissues were finally embedded in pure Spurr resin and polymerized at 70°C for 9 h. Gold sections were prepared on a Reichert-Jung ultra-microtome, collected onto formvar coated grids and stained with lead citrate and uranyl acetate. Observations were made using a Philips 201 transmission electron microscope (Eindhoven, The Netherlands) or a Philips Tecnai 12 transmission electron microscope (Philips Electron Optics, Eindhoven, Netherlands) operated at 80 kV and fitted with a Kodak (Rochester, New York, USA) Megaview II camera with software (AnalySIS, Soft Imaging System, Münster, Germany).

### Terminal Deoxynucleotidyl Transferase-Mediated dUTP Nick End Labeling Assay

Tissue pieces approximately 5 mm^2 ^were excised from window stage leaves and fixed in FAA for 2 h, followed subsequently by 3 washes in phosphate buffered saline (PBS). The terminal deoxynu-cleotidyl transferase-mediated dUTP nick end labeling (TUNEL) assay was performed according to the manufacturer's instructions (Roche Di-agnostics, Mannheim, Germany). Nuclei were counterstained by incubation in 3% (w/v) propidium iodide for 2 min. Samples were observed via confocal microscopy. A negative control was performed without the terminal deoxynucleotidyl transferase enzyme, and a positive control was performed with DNase1.

### Mitochondrial Staining

Tissue pieces approximately 5 mm^2 ^were excised from window stage leaves and stained for one hour in 0.2 μM, 0.3 μM, 0.5 μM, 0.6 μM, 1 μM, and 2 μM CMXRos (Invitrogen, Eugene, OR, USA; dissolved in dimethylsulfoxide, DMSO). Leaf sections were then rinsed with ddH_2_0 eight times and shaken for 90 minutes in ddH_2_0 at approximately 100 rpm. Leaf sections were then mounted on slides and excited with the TRITC cube (excitation 527-552 nm and emission 577-632 nm) to view mitochondrial fluorescence and the FITC cube (excitation 460-500 nm emission 510-560 nm) to view the corresponding chlorophyll autofluorescence using the confocal microscope.

These four stages of mitochondrial dynamics (M1-M4) were consistent within a window stage leaf when viewing the surface of epidermal cells where mitochondria are pushed up against the plasma membrane. However, when looking deeper into an epidermal cell, where mitochondria are found within the thin ring of cytoplasm between the plasma membrane and tonoplast membrane, due to the differing focal plane and orientation, mitochondrial dynamics can vary in appearance. It was important therefore, that for quantitative measurements, that the videos and images were taken from the very top portion of epidermal cells. Mitochondrial staining was completed at least 15 times.

### Cyclosporine A treatment

Healthy plants between 3 to 4 weeks of age, containing at least 2 perforated leaves, were selected for use in CsA trials. Plants were divided at random into experimental or control groups and under sterile conditions, liquid medium was poured out of each magenta box and replaced with 200 mL of fresh liquid medium. For the treatment groups, CsA stock dissolved in 90% ethanol was added to the liquid medium to make a final concentration of 2 μM, 4 μM, 10 μM, 15 μM, or 20 μM CsA in the boxes. For control plants, an equivalent volume of ethanol was added to the liquid medium. The plants were then returned to the growth racks under normal light conditions until they were at the proper stage for harvesting. Digital photographs acquired with a Nikon Coolpix P5000 camera (Nikon Canada Inc., Mississauga, ON, Canada) were taken of each plant for each concentration at least twice a week in order to track growth of newly emerging leaves. For the examination of mitochondria in leaves that had been treated with CsA, CMXRos staining was carried out as described above.

### Harvesting plants

Plants were considered ready to harvest following approximately 3 to 4 weeks from the initiation of the inhibitor experiment. Using the successive images, which were taken each week, and labeled by means of Adobe Photoshop Elements 6, version 6.0, each leaf was identified and removed from the respective magenta box. The petiole of each leaf was cut to 1 cm in length and the number of perforations per leaf was counted as an indicator of PCD. Leaf length was also measured as an indicator of normal leaf development. Number of perforations in an individual leaf was then divided by individual leaf length to obtain the variable 'ratio of number of perforations per cm of leaf length'. This variable is a more inclusive measure of PCD than solely number of perforations due to it accounting for the assumption that number of perforations depends in part on leaf length. Each leaf was then individually blotted dry, flattened by hand, and aligned in chronological order of emergence for photography purposes.

### Statistical analysis

Data were assessed by a general linear model of variance (GLM ANOVA) and the means were compared by the Tukey test at 95% confidence intervals (P < 0.05). Statistical analyses were carried out using Minitab 15 Statistical Software English (Minitab Inc., State College, PA, USA).

## Authors' contributions

CENL and JW carried out all experiments: pharmacological application and harvest, light, fluorescent, confocal and DIC microscopic observations and measurements, along with conceptual mitochondrial staging. CENL completed the statistical analysis, drafted and revised the final manuscript. JW also contributed to final manuscript and completed the revisions. SL completed transvacuolar strand observations using light microscopy, and live cell-imaging. AHLANG conceived the study, participated in its design and coordination, helped in drafting and revising the manuscript, and supervised all experimental work. All authors read and approved the final manuscript.

## Supplementary Material

Additional file 1 **Stage M1 mitochondrial dynamics**. CMXRos stained NPCD cell, highlighting stage M1 mitochondrial dynamics. Note individual mitochondria actively streaming within the cytosol. Click here for file

Additional file 2 **Stage M2 mitochondrial dynamics**. CMXRos stained EPCD cell, highlighting stage M2 mitochondrial dynamics. Note the aggregation of mitochondria along with several individual mitochondria, all of which appear to be moving. Click here for file

Additional file 3 **Stage M2-M3 mitochondrial transition**. CMXRos stained EPCD cell and DIC overlay. Video highlights the transition from stage M2 to stage M3 mitochondria. Note mitochondrial aggregate moving towards the nucleus, followed by cessation of movement. Video is 15× normal speed. Click here for file

Additional file 4 **Stage M3 mitochondrial dynamics**. CMXRos stained LPCD cell, highlighting stage M3 mitochondrial dynamics. Note the absence of movement of the mitochondrial aggregate. Click here for file

Additional file 5 **Stage M4 mitochondrial dynamics**. CMXRos stained LPCD cell, aimed at highlighting stage M4 mitochondrial dynamics. Note the lack of mitochondrial staining by CMXRos possibly due to complete loss of ΔΨ_m_. Click here for file

Additional file 6 **Transvacuolar strands in NPCD stage cells**. NPCD stage cells showing several transvacuolar strands, and highlighting the close association and possible movement of mitochondria and chloroplasts along them. Video 20× normal speed Click here for file

Additional file 7 **Transvacuolar strands in EPCD stage cells**. EPCD stage cells showing increased transvacuolar strands activity and highlighting the close association and possible movement of mitochondria and chloroplasts along them. Note the trajectory of most strands and organelles towards the nucleus. Video 20× normal speed Click here for file

Additional file 8 **Transvacuolar strands in LPCD stage cells**. LPCD stage cells showing a decrease in the number of transvacuolar strands and absence of organelles. Note that although mitochondrial streaming in often ceased at this point, slight cytoplasmic streaming can be visualized in mesophyll cells below the point of focus. Video 20× normal speed. Click here for file

Additional file 9 **Mitochondrial dynamics in CsA treated NPCD stage cells**. CsA treated leaf subsequently stained with CMXROS, depicting a single cell that corresponds with an NPCD window stage cell. Note, individual mitochondria that are rapidly moving within the cytosol. Click here for file

Additional file 10 **Mitochondrial dynamics in CsA treated EPCD stage cells**. CsA treated leaf subsequently stained with CMXROS, depicting a single cell that corresponds with an EPCD window stage cell. Note, individual mitochondria that are rapidly moving in the cytosol. Click here for file

Additional file 11 **Mitochondrial dynamics in CsA treated LPCD stage cells**. CsA treated leaf subsequently stained with CMXROS, depicting a single cell that corresponds with an LPCD window stage cell. Note, many individual mitochondria, and several small aggregates that are rapidly moving. Click here for file
