# Correction: Do mitochondria play a role in remodelling lace plant leaves during programmed cell death?

**DOI:** 10.1186/1471-2229-13-58

**Published:** 2013-04-02

**Authors:** Christina EN Lord, Jaime N Wertman, Stephanie Lane, Arunika HLAN Gunawardena

**Affiliations:** 1 Department of Biology, Dalhousie University, 1355 Oxford Street, Halifax, B3H 4R2, Canada

## Correction

Following the publication of this work [1] we became aware of errors in image selection from introductory Figure one. Figure one C [1] had been previously published as Figure one C in [2]; additionally Figure one F [1] had been previously published as Figure one E in [3]. This duplication of figures was inadvertent and we have now received permission from both publishers (Global Science Books [2] and Elsevier [3]) to reproduce these images. Note that these figures were for introductory demonstration purposes only, and this correction does not affect the scientific content of the article. We would like to apologize for any confusion or inconvenience this may have caused.
